# Machine Learning Methods for Predicting Postpartum Depression: Scoping Review

**DOI:** 10.2196/29838

**Published:** 2021-11-24

**Authors:** Kiran Saqib, Amber Fozia Khan, Zahid Ahmad Butt

**Affiliations:** 1 School of Public Health Sciences University of Waterloo Waterloo, ON Canada

**Keywords:** machine learning, postpartum depression, big data, mobile phone

## Abstract

**Background:**

Machine learning (ML) offers vigorous statistical and probabilistic techniques that can successfully predict certain clinical conditions using large volumes of data. A review of ML and big data research analytics in maternal depression is pertinent and timely, given the rapid technological developments in recent years.

**Objective:**

This study aims to synthesize the literature on ML and big data analytics for maternal mental health, particularly the prediction of postpartum depression (PPD).

**Methods:**

We used a scoping review methodology using the Arksey and O’Malley framework to rapidly map research activity in ML for predicting PPD. Two independent researchers searched PsycINFO, PubMed, IEEE Xplore, and the ACM Digital Library in September 2020 to identify relevant publications in the past 12 years. Data were extracted from the articles’ ML model, data type, and study results.

**Results:**

A total of 14 studies were identified. All studies reported the use of supervised learning techniques to predict PPD. Support vector machine and random forest were the most commonly used algorithms in addition to Naive Bayes, regression, artificial neural network, decision trees, and XGBoost (Extreme Gradient Boosting). There was considerable heterogeneity in the best-performing ML algorithm across the selected studies. The area under the receiver operating characteristic curve values reported for different algorithms were support vector machine (range 0.78-0.86), random forest method (0.88), XGBoost (0.80), and logistic regression (0.93).

**Conclusions:**

ML algorithms can analyze larger data sets and perform more advanced computations, which can significantly improve the detection of PPD at an early stage. Further clinical research collaborations are required to fine-tune ML algorithms for prediction and treatment. ML might become part of evidence-based practice in addition to clinical knowledge and existing research evidence.

## Introduction

### Background

Postpartum depression (PPD) is considered one of the most frequent maternal morbidities after delivery, with severe implications for the mother and child. According to the National Institute of Mental Health, United States, 10%-15% of women have maternal depression during and after pregnancy worldwide, whereas in low- and middle-income countries, this percentage could be as high as 18%-25% [1] and seems to depend on the cultural and traditional characteristics of the population [2]. Both the Diagnostic and Statistical Manual of Mental Disorders (DSM-IV) and the International Classification of Diseases (ICD)-10 recognize maternal depression as a mental illness with different classifications [3].

PPD, the most common complication of childbearing, is a term applied to depressive symptoms that occur within 4 weeks of giving birth and possibly as late as 30 weeks postpartum [4]. PPD is a significant public health issue that affects women as well as child’s physical and mental health and cognitive and interactive development [5], thus making the child vulnerable to developing psychiatric disorders during adolescence [6]. A depressed mother may not establish a positive relationship with her infant [7], and this may continue to affect children into toddlerhood, preschool years, and beyond [8]. Infants of depressed mothers have shown poor nutrition, poor general health, and more frequent diarrheal episodes, and in extreme cases, maternal suicide and infanticide may occur [9,10]. PPD is generally an overlooked health problem that can lead to serious complications and should be addressed in a timely manner [11].

As there is no single etiology for PPD, a single prevention method or treatment will be ineffective. There is a need for a multifactorial approach combining psychological, psychosocial, and biological predictive factors of PPD to contemplate various etiological factors and individual variations [12,13]. An effective PPD prediction model can help health care providers in the early identification and effective management of at-risk patients [14], with evidence from previous studies exploring this possibility and feasibility [15].

Machine learning (ML) algorithms are broadly grouped into 3 categories: (1) supervised, (2) unsupervised, and (3) semisupervised learning. In supervised learning, data with known labels are used to train a model that can predict the label for new data [16]. ML-based predictive models are gaining popularity for combining a huge amount of data into a single model and evaluating the model’s predictive value for previously unseen individuals, for example, at-risk and new patients. ML approaches rely on the use of advanced statistical and probabilistic techniques to construct systems with the ability to automatically learn from data. This enables patterns in data to be more readily and accurately identified and more accurate predictions to be made from data sources (eg, more accurate diagnosis and prognosis) [17]. ML has been used for prediction in psychiatry [18]. ML methods have been successfully used to predict major depressive disorder persistence, chronicity, severity [19], and treatment response [20]. The key to building good ML models is in the rigorous selection of appropriate features and algorithms [17]. Recently, a scoping review of ML application in mental health identified over 190 studies that applied ML in the detection and diagnosis of mental disorders and over 60 studies to predict the progression of mental health problems over time [21]. These studies reported the use of electronic health records (EHRs), mood rating scales, brain imaging data, smartphone monitoring systems, and social media platforms to predict, classify, or subgroup mental health illnesses, including depression, schizophrenia, and suicide ideation and attempts [22]. Two main ML algorithms have been commonly reported in depression prediction studies, namely, support vector machine (SVM) and random forest (RF) algorithms [21]. Depression prediction studies using these 2 methods have achieved relatively good results [23-25].

There is an opinion that ML will help mental health practitioners redefine mental illnesses more objectively than is currently done in the Diagnostic and Statistical Manual of Mental Disorders [3] and would help in the early identification of these illnesses to make interventions more effective [22]. Thus, in addition to disease-model refinement, ML may benefit psychiatry by characterizing those at risk and personalizing and discovering pharmacological therapeutics [26,27].

A literature review of ML and big data research analytics in maternal depression is pertinent and timely, given the rapid technological developments in recent years. This review aims to provide a concise snapshot of the literature on ML applications for predicting PPD. Previous reviews have demonstrated ML techniques to be robust and scalable for general depression and mental health, but no review to date has mapped ML applications within maternal mental health research and practice. Our overall aim is to examine the current state of affairs of ML applications in PPD, providing a snapshot of the methods used. Keeping in view the rapid advancements in ML and the recent use of ML in mental health research, we chose to focus specifically on exploring broadly the nature of research activity, as per the first goal of scoping reviews by Arksey and O’Malley [28].

### Objective

It is hoped that this scoping review will (1) inform mental health researchers of the methods and applications of ML in the context of prediction of PPD, (2) identify the best-performing algorithm, and (3) identify the evaluation criteria for the best-performing algorithm.

## Methods

### Overview

The Arksey and O’Malley framework was used in addition to methodological improvements for scoping review [28-30]. Our methods also align with the PRISMA-ScR (Preferred Reporting Items for Systematic Reviews and Meta-Analyses extension for Scoping Reviews) checklist [31]. A scoping review methodology was chosen to map the body of literature on the use of ML in predicting PPD, including a greater range of study designs and methodologies, to provide a descriptive overview of the reviewed material.

### Search Strategy

The search strategy was adapted from Shatte et al [21], which is a similar review of big data applications in mental health. As ML and PPD stretch across interdisciplinary fields, the search was conducted in both health and information technology databases. First, a literature search was conducted using health-related research databases, including PsycINFO and PubMed. Next, the information technology databases IEEE Xplore and the ACM Digital Library were searched. Finally, databases that index both fields, including Scopus and Web of Science, were searched. The search period for relevant studies was conducted in September 2020. The search terms included variations in the terms for the following:

- (a) PPD (*maternal∗, perinatal∗, postpartum blues∗, baby blues∗, depression∗, post birth depression∗*)
- (b) ML (machine learning*, artificial intelligence*, supervised learning*, big data*)
- (c) Prediction (predictive models∗, prediction∗, detection*)

The search was conducted on titles, keywords, and abstracts with *AND* entered into the database search to link different categories (a, b, and c) of search terms. Truncation symbols (∗) were used to search for all possible forms of a search term (Multimedia Appendix 1). Forward reference searching, that is, examining the references cited in these articles, and backward reference searching, that is, reviewing the references cited in these articles, were applied to identify further studies that met the inclusion criteria.

### Study Selection

Articles were included and excluded (Textbox 1) in the review if the following criteria were met.

**Inclusion and exclusion criteria.**

**Inclusion criteria**

- The article reported on a method or application of machine learning (ML) to address postpartum depression only, based on the authors’ descriptions of their analyses: if they deemed it ML, the paper was included.
- The article evaluated the performance of the ML algorithm or big data technique used to predict postpartum depression.
- The article was published in a peer-reviewed publication.
- The article was available in English.
- The article was published between 2009 and 2021.

**Exclusion criteria**

- The article did not report ML applications in postpartum depression (eg, the paper commented on the use of ML in diagnosis, treatment, or prognosis of general depression, anxiety, and other mental health issues).
- The article did not focus on postpartum depression.
- The full text of the article was not available (eg, conference or abstracts).
- If articles were commentaries and essays. Two reviewers (KS and AFK) independently reviewed all studies and reached a consensus on all included studies after consultation with the third author (ZAB).

### Data Extraction and Analysis Plan

For data extraction and analysis, we used the same framework already used in a similar scoping review [32]. For each article, data were extracted regarding (1) overall aim of research, that is, prediction and area of focus, that is, PPD; (2) input data type used; (3) type of ML algorithms used; and (4) the best-performing algorithm, that is, results.

To analyze the data, a narrative review synthesis method [32] was selected to capture the extensive range of research investigating ML and big data for PPD prediction. A meta-analysis was not deemed appropriate, given the aim of identifying research activity in the interdisciplinary field of big data and maternal mental health.

## Results

### Overview

**Figure 1:**
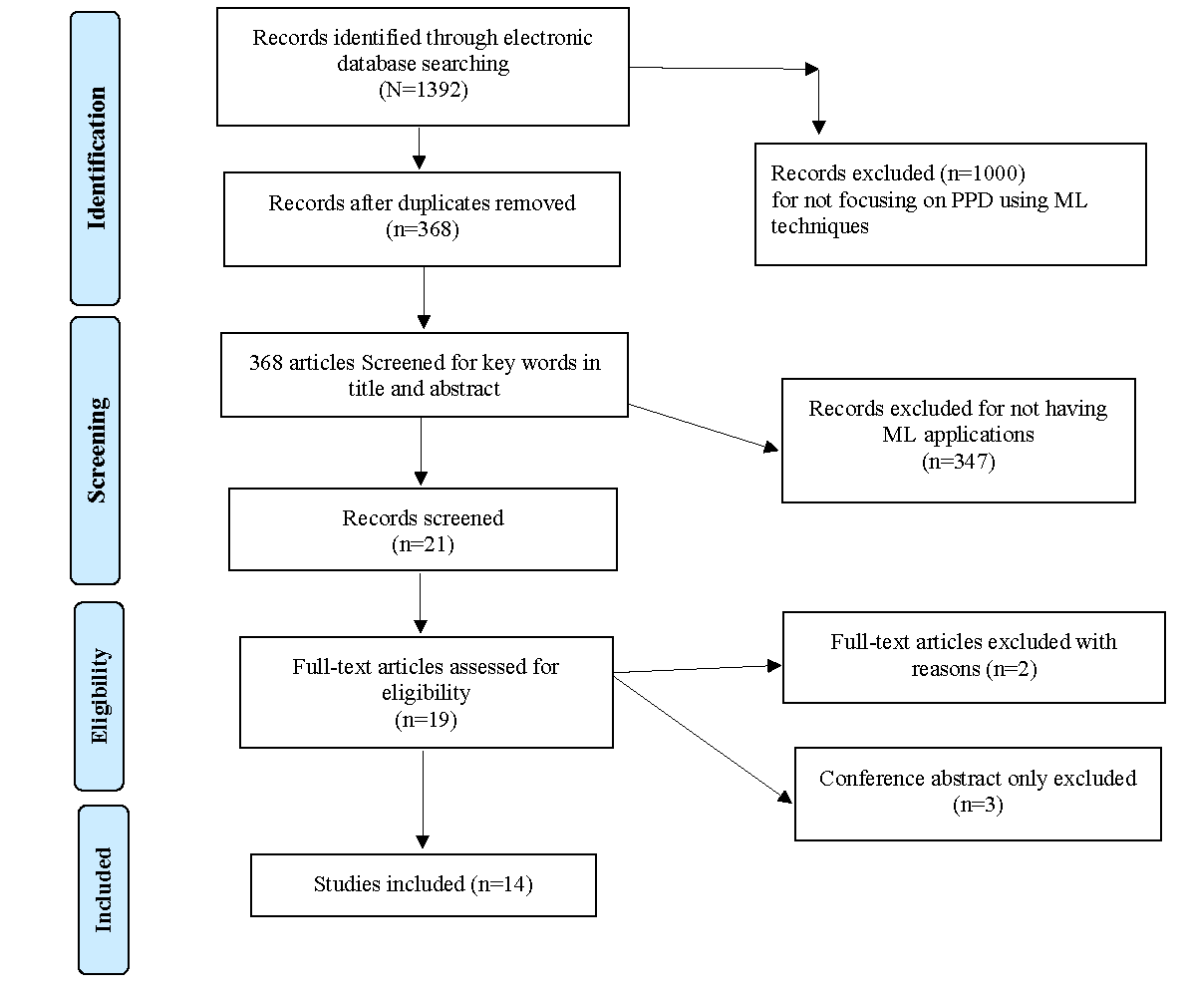
PRISMA (Preferred Reporting Items for Systematic Reviews and Meta-Analyses) procedural flowchart. ML: machine learning; PPD: postpartum depression.

The search strategies using a combination of search terms identified 1392 articles that included a search term from each category in their abstract or title (PRISMA-ScR flowchart). The range for publication year of relevant articles was 2009-2021. A total of 24 articles were duplicates. A database search was carried out by KS and AFK. Abstracts of 368 articles were read by both authors to perform an initial screening of eligibility for this scoping review. Of these, 347 were excluded because they did not focus specifically on PPD. A total of 21 articles were selected for full-text review, but 3 were conference papers and abstract only, and 4 did not use ML to predict PPD. This resulted in a total sample of 14 studies, including one preprint and one focused on predicting PPD in fathers, which met the inclusion criteria according to all authors (Figure 1). The selected 14 studies were reviewed in full by 2 authors (including KS and AFK). A mutual consensus was reached after the final approval from ZAB. In the subsequent narrative analysis, we focus on the 14 studies that reported using the ML model to predict PPD (see Tables 1 and 2 for a summary of the main study characteristics).

**Table 1:** Summary of the main study characteristics (N=14).

#	Study	Aims or objectives	Sample size; input data used	Diagnosis criteria for PPD^a^
1	Jiménez-Serrano et al [24]	Develop classification models for detecting the risk of PPD during the first week after childbirth	1880; hospital data	EPDS^b^>9; 8th or 32nd week postbirth
2	Betts et al [33]	Develop a prediction model to identify women at risk of postpartum psychiatric admission	75,054; linked administrative health data	ICD^c^-10 (*F*_20.0_-*F*_39.9_) or ICD-10: (*F*_53.0_-*F*_53.1_)
3	Tortajada et al [34]	To obtain a classification model based on feedforward multilayer perceptron to improve PPD prediction during the 32 weeks after childbirth with a high sensitivity and specificity	1397; hospital data	EPDS>9; 8th or 32nd week postbirth
4	Wang et al [35]	To develop a PPD prediction model, using EHRs^d^	179,980; EHRs	ICD-10-CM codes O99.3 and O99.34 as well as their ICD-9-CM equivalents for a diagnosis of PPD within 12 months after childbirth
5	Zhang et al [36]	To compare the effects of 4 different ML^e^ models using data during pregnancy to predict PPD	508; hospital data	EPDS >9.5; within 42 days postdelivery
6	Zhang et al [37]	Propose an ML framework for PPD risk prediction	17,633 and 71,106; 2 data sets from EHRs	PPD within 1 year of childbirth
7	Hochman et al [38]	To apply ML approach to create a prediction tool for PPD to be implemented in health care systems	214,359; EHRs	PPD within first year postpartum (ICD‐9 codes: 300 and 309 or ICD-10 codes: F40-F48) or acute psychotic manic episodes (ICD‐9 codes: 296.0, 296.1, 296.4, 296.6, 296.81, 298.3, 298.4, 298.8)
8	De Choudhury et al [39]	Detect and predict PPD	165; Facebook survey using PHQ^f^-9	PHQ-9
9	Natarajan et al [23]	Propose an ML-based approach for PPD prediction and diagnosis from survey information	207; Facebook and Twitter survey data	Postpartum Depression Predictors Inventory
10	Fatima et al [40]	Use linguistic features to propose a solution for PPD that can be generalized and deployed across web-based social platforms	21; text posts from Reddit	PPD based on linguistic feature
11	Trifan et al [41]	To use social media for potential diagnosis of mothers at risk of PPD and thus the implementation of early interventions	512; Reddit text posts	Not described
12	Shatte et al [42]	To identify fathers at the risk of PPD	365; Reddit text posts	ICD-10 depression; symptom 06 months postbirth
13	Moreira et al [43]	Propose an algorithm for emotion-aware smart systems, capable for predicting the risk of PPD during pregnancy through biomedical and sociodemographic data analysis	Performance evaluation used data generated by wearable devices and sensors	Not described
14	Shin et al [44]	To develop predictive models for PPD using ML approaches	28,755; pregnancy risk assessment and monitoring system data	PHQ-2

^a^PPD: postpartum depression.

^b^EPDS: Edinburgh Postnatal Depression Scale.

^c^ICD: International Classification of Diseases.

^d^EHR: electronic health record.

^e^ML: machine learning.

^f^PHQ: Patient Health Questionnaire.

**Table 2:** Summary of the main study characteristics (N=14).

#	Study	Performance metric	ML^a^ algorithms used	Best-performing algorithm
1	Jiménez-Serrano et al [24]	Hold-out validation	Naive Bayes LR^b^ SVM^c^ ANN^d^	Naive Bayes model; G function value of 0.73
2	Betts et al [33]	5-Fold cross-validation in R	Gradient boosting Elastic net methods	Boosted trees algorithm (AUC^e^ 0.80, 95% CI 0.76-0.83)
3	Tortajada et al [34]	Hold-out validation	ANN	Multilayer perceptrons 0.82 of G and 0.81 of accuracy (95% CI 0.76-0.86) with 0.84 of sensitivity and 0.81 of specificity
4	Wang et al [35]	10-fold cross-validation	SVM RF^f^ Naive Bayes L2-regularized LR XGBoost^g^ DT^h^	SVM with AUC (0.79)
5	Zhang et al [36]	sklearn.cross_validation package in Python	SVM RF	SVM and feature selection RF (sensitivity=0.69; AUC=0.78)
6	Zhang et al [37]	5-Fold cross-validation	RF DT XGboost Regularized LR Multilayer perceptron	LR with L2 regularization; AUC (0.937, 95% CI 0.912-0.962)
7	Hochman et al [38]	Hold-out cross-validation	XGBoost	AUC of 0.712 (95% CI 0.690-0.733), with a sensitivity of 0.349 and a specificity of 0.905)
8	De Choudhury et al [39]	Not described	Regression models to develop a series of statistical models	Postnatal model
9	Natarajan et al [23]	Information not provided	Functional gradient boosting DT SVM NB^i^	Functional gradient boosting (Roc) 0.952
10	Fatima et al [40]	10-Fold cross-validation	LR SVM Multilayer perceptron	Multilayer perceptron; 91∙7% accuracy for depressive content identification and up to 869% accuracy for PPD content prediction
11	Trifan et al [41]	Hold-out validation	SVM Stochastic gradient descent Passive aggressive classifiers	SVM
12	Shatte et al [42]	10-Fold cross-validation	SVM classifiers using behavior, emotion, linguistic style, and discussion topics as features	0.67 precision, 0.68 recall, and 0.67F−measure in model including all features
13	Moreira et al [43]	10-fold cross-validation	DT SVM Nearest neighbor Ensemble classifiers	Ensemble classifiers
14	Shin et al [44]	10-Fold cross-validation	RF Stochastic gradient boosting SVM Regression trees NB k-nearest neighbor LR ANN	RF method (AUC) 0.884

^a^ML: machine learning.

^b^LR: logistic regression.

^c^SVM: support vector machine.

^d^ANN: artificial neural network.

^e^AUC: area under the curve.

^f^RF: random forest.

^g^XGBoost: Extreme Gradient Boosting.

^h^DT: decision tree.

^i^NB: Naive Bayes.

A narrative synthesis of ML activity, particularly in the context of PPD, indicated the emerging nature of this field, with most studies being published in recent years. Publication dates ranged from 2009 to 2020; however, most articles were very recent. There is a 5-year gap between the first 2009 article [34] and the next study in 2014 [39], and publications have accelerated recently with 7 papers published in 2020.

Few studies have focused on developing and testing an ML algorithm for the detection and prediction of PPD, whereas other studies focused on comparing the effects of different ML algorithms to predict PPD and explore which factors in the model are the most important for PPD prediction.

### Type of Input Data

When we examined the 14 studies, we identified a subgroup of 7 studies that reported on the use of ML-based models to predict PPD using clinical or hospital data and EHRs. The other 5 studies reported on the application of ML algorithms for the prediction of PPD using data from social media platforms, including Facebook, Twitter, and Reddit. However, these studies were designed to evaluate a prediction model more broadly and did not report details on ML algorithms, training, and testing procedures. Of the remaining 2 studies, one reported on the use of population data and the other used emotion-aware system data. The outcome variable *PPD* was assessed using psychometric tools such as Patient Health Questionnaire-9, Patient Health Questionnaire-2, Edinburgh Postnatal Depression Scale, Postpartum Depression Predictors Inventory, and ICD-9 and ICD-10 codes in the case of hospital and EHR data, whereas linguistic features were used to predict PPD from text data of social networks.

### Type of ML Algorithms Used

All studies reported on the use of supervised ML models, including classification and regression algorithms, to predict PPD. Most of the studies (n=7) reported using more than one algorithm, whereas one study used only regression models to develop statistical models for their data. These included SVM (n=8) logistic regression (LR; n=6), multilayer perceptron using artificial neural network (ANN; n=5), RF (n=4), Naive Bayes (n=3), decision trees (DTs; n=3), gradient boosting (n=2), XGBoost (Extreme Gradient Boosting; n=2), functional gradient boosting (n=1), elastic net methods (n=1), k-nearest neighbor (kNN; n=2), Stochastic Gradient Boosting (n=1), passive aggressive classifiers (n=1), and ensemble classifier (n=1). The data types used to develop ML algorithms included EHRs, either administrative hospital data or organizational data (n=08), mobile and wearable sensor data (n=1), and social media data (n=5).

### Reported Best-Performing Algorithm

There was considerable heterogeneity in the best-performing ML algorithm across the selected studies. To report the best-performance algorithm, most studies used sensitivity, specificity, and area under the curve (AUC). Only 5 studies described the technical approaches to cross-validation using either 5-fold or 10-fold cross-validation. One study reported that of 4 ML algorithms, including Naive Bayes, LR, SVM, and ANN, Naive Bayes showed the best balance between sensitivity and specificity as a predictive model for PPD during the first week after delivery according to the G function, with a value of 0.73 [24]. Another study using 6 ML models, including SVM, RF, Naive Bayes, L2-regularized LR, XGBoost, and DT, reported that SVM had the best performance, and the difference across the performance of SVM, L2-regularized LR, RF, Naive Bayes, and XGBoost was minimal, although differences existed with respect to sensitivity and specificity [35]. In total, 9 different ML algorithms, including RF, stochastic gradient boosting, SVM, recursive partitioning and regression trees, Naive Bayes, kNN, LR, and neural network, were used to report the overall classification accuracies of the 9 models ranging from 0.650 (kNN) to 0.791 (RF). The RF method achieved the highest area under the receiver operating characteristic curve (AUROC) value of 0.884, followed by SVM, which achieved the second-best performance with an AUC value of 0.864 [44].

Using the SVM and RF algorithms, the model based on SVM and feature selection RF had the best prediction effects (sensitivity=0.69, AUC=0.78) [36]. Five ML algorithms were trained: RF, DT, XGBoost, regularized LR, and multilayer perceptron. LR with L2 regularization was found to be the best-performing algorithm using data available up to childbirth. The AUC was 0.937 (95% CI 0.912-0.962) and 0.886 (95% CI 0.879-0.893) in hospital data sets, respectively [37]. SVM led to slightly better results in terms of F1 in the validation stage compared with stochastic gradient descent and passive aggressive classifiers [41].

Tortajada et al [34] developed 4 models for predicting PPD using a multilayer perceptron and evaluated them with the geometric mean of accuracies using a hold-out strategy. They reported that the developed models could predict PPD during the first 32 weeks after delivery with high accuracy. A similar study reported that hold-out validation showed that multilayer perceptron outperformed other techniques such as SVM and LR used in one study with 91.7% accuracy for depressive content identification and up to 86.9% accuracy for PPD content prediction [40]. Another study using gradient boosting and elastic net methods reported that the boosted trees algorithm produced the best-performing model, predicting postpartum psychiatric admission in the validation data with good discrimination (AUC 0.80, 95% CI 0.76-0.83) and achieved good calibration. This model outperformed the benchmark LR model and the elastic net model [33]. Natarajan et al [23] reported a successful functional gradient boosting algorithm that demonstrated the potential of ML in predicting PPD.

Hochman et al [38] built a model using XGBoost, an algorithm based on gradient-boosted DTs, and assessed the overall model predictive performance using the AUROC. 95% CIs were estimated using bootstrapping. The prediction model achieved an AUC of 0.712 (95% CI 0.690-0.733), with a sensitivity of 0.349 and a specificity of 0.905 at the 90th percentile risk threshold, identifying PPDs at a rate more than 3 times higher than the overall set (positive and negative predictive values were 0.074 and 0.985, respectively).

After developing a series of statistical models using regression models to predict a mother’s likelihood of PPD, the postnatal model performed the best [39]. Predictive models were developed as a series of SVM classifiers using behavior, emotion, linguistic style, and discussion topics as features. The model incorporating behavior and discussion topic features alone yielded greater recall, with 0.77 and 0.82, respectively, which may be useful for screening purposes [42]. A study using hospital data showed that ensemble classifiers represent a leading solution for predicting psychological disorders related to pregnancy [43].

Many studies did not mention which statistical tools were used for analysis; however, most used a variety of software packages in R, SAS, and Python 3. Studies have reported the use of standard libraries available for data preparation (eg, missing variables), a variety of typical ML models, and natural language processing (NLP) analyses (such as topic modeling) included in their standard packages such as R.

## Discussion

### Principal Findings

Most of the reviewed studies used supervised classification techniques rather than other ML techniques to predict PPD. This is perhaps indicative of the extensive focus on detection and diagnosis in the literature, which is typically designed using large, retrospective, labeled data sets ideal for classification tasks [45]. All reviewed studies concluded that ML models were effective in predicting PPD, whether clinical data, EHRs, population data, and data from social media platforms. All the studies implied that the ML approach was more beneficial compared with traditional statistical approaches. However, the level of accuracy, sensitivity, or specificity that is considered acceptable varies depending on the aims of the study and the data set. None of the studies explicitly compared the ML performance with other traditional statistical analyses. In all studies, the ML approach aided researchers in answering their research questions.

The results from a cohort study for predicting PPD using hospital data reported that in the case of a small sample size, SVM can avoid overfitting while providing efficient computing time and better prediction results in depression [46,47]. The same study proposed that when the data set is small, SVM is more practical than RF in prediction research for PPD [36]. Several previous studies used the SVM algorithm to make PPD predictions, as SVM is an example of supervised learning that is most commonly used in classification problems. It focuses on minimizing the structural risks within a set of available data [36]. It has significant advantages and performs well in situations with relatively less available sample data [48]. SVM is a classifier that transforms input data into a multidimensional hyperplane using kernels to discriminate between 2 classes [49]. Jiménez-Serrano et al [24] collected data on postpartum women from 7 Spanish hospitals and used the Edinburgh Postnatal Depression Scale score as the outcome indicator to train a PPD prediction model based on SVM. Natarajan et al [23] used social media as a data source, and based on the mental health data of 173 mothers, an SVM-based PPD prediction model was established. De Choudhury [39] developed an SVM model to identify high-risk emotions and behaviors predictive of PPD using the content of Twitter posts. As these studies either target different populations or use different methods to detect the occurrence of PPD, the model prediction effects cannot be easily compared [36].

In contrast, RF models were built using a DT as the basic classifier. RF approaches have high classification accuracy, strong inductive capacity, a simple parameter adjustment process, fast calculation speed, relatively low sensitivity to missing data values, and the ability to output feature importance [50,51]. RF is an ensemble learning method that operates by constructing a multitude of DTs and outputting the class that is voted by a majority of the trees [52], and Shin et al [44] reported RF to be the best-performing algorithm for predicting PPD.

Tortajada et al [34] developed another prediction model for PPD using multilayer perceptron and pruning for pregnant Spanish women using data from 7 Spanish general hospitals from 2003 to 2004. ANNs have a remarkable ability to characterize discriminating patterns and derive meaning from complex and noisy data sets. They have been widely applied in general medicine for the differential diagnosis, classification, prediction of disease, and condition prognosis. For instance, ANNs have been applied to the diagnosis of dementia using clinical data [53] and more recently for predicting Alzheimer disease using mixed effects neural networks [54].

There is a great deal of debate about which ML model evaluation metric is best [55]. Making sense of reported ML evaluation metrics is made even more difficult because different performance parameters often provide conflicting results and the optimal ML algorithm also depends significantly on the composition of the data set [56]. Some reviewed studies reported varying degrees of accuracy and were not always explicitly clear regarding the meaning of the resulting performance metrics. Owing to the negative effects of PPD on mothers and infants [57,58], such as the negative effects on the physical and mental health of mothers, the closeness of the mother-infant bond, and infant development, it is important to have a model with high sensitivity while maintaining a high AUROC value. The selection of indicators for evaluating depression prediction models varies across studies. For example, Natarajan et al [23] and De Choudhury [39] emphasized the accuracy of the model’s prediction of PPD. Jiménez-Serrano et al [24] emphasized the sensitivity and specificity of the model. The balance between the two is the geometric mean. The AUROC is also widely used to evaluate the comprehensive performance of a model [23,25].

PPD is a highly prevalent problem but frequently goes undetected, leading to substantial treatment delays [59]. EHRs collect a large number of biometric markers and patient characteristics that could foster the detection of PPD in primary care settings. NLP and ML have the potential to complement clinical practice by categorizing and analyzing data from clinical notes [60]. NLP is a computerized process that analyzes and codes human language into text [61] that ML algorithms can analyze and use to predict outcomes [62]. Advances in technology, such as social media, smartphones, wearables, and neuroimaging, have allowed mental health researchers and clinicians to collect a vast range of data at a rapidly growing rate [63]. ML is a vigorous technique with the ability to analyze these data. A data-driven primary intervention approach using ML and EHR data may be leveraged to reduce the burden of health care providers in identifying PPD risk [37].

In the studies included in our review, individuals experiencing PPD were identified through screening surveys, their public sharing of a diagnosis on social media, Twitter, Facebook, or Reddit, and were distinguishable from control users by patterns in their language and web-based activity [23,40,42]. Automated detection methods may help identify depressed or otherwise at-risk individuals through the large-scale passive monitoring of social media and, in the future, may complement existing screening procedures [64]. Social media data and EHRs both hold the promise of innovating in the maternal mental health domain, particularly when leveraged by ML techniques [21].

Finally, there are some challenges to consider when using ML techniques in mental health applications. ML models are inevitably limited by the quality of the data used to develop the model. As such, ML does not replace other research or analytic approaches; rather, it has the potential to add value to mental health research. Many ML techniques require access to training data sets, which calls for collaboration between researchers and clinicians to maximize the usefulness of the models developed. It is important to highlight that ML might become part of evidence-based practice, in addition to clinical knowledge and existing research evidence. Greater collaboration between mental health researchers and clinicians (eg, for the provision of training data sets and for feedback on the clinical usefulness of ML algorithms) will be needed to continue to advance the applications of ML in mental health. Analyzing *big data* on clinical outcomes, in addition to genetic, biomedical, behavioral, environmental, and demographic patient characteristics, could help predict maternal depression. EHR databases can provide valuable, real-world, practice-based evidence to support better prediction models for at-risk patients [65]. In this way, ML offers a solution for analyzing idiographic research questions in big data [66].

### Limitations

This study has a few limitations. The aim of this scoping review was to provide a snapshot of the research activity in a summarized format while using a systematic search method. In line with the aims of a scoping review, we did not identify specific study designs in advance and did not assess the quality of the included studies [28]. Moreover, because of restrictions in the search methodology, there may be a chance to have missed some relevant articles, for example, broad search terms and the exclusion of nonpeer reviewed literature. This is a common limitation reported in scoping review studies attributed to maintaining a balance between breadth and depth of analysis within a rapid timeframe [67]. This review successfully mapped a cross-section of the literature on the use of ML for PPD prediction and provides a useful synthesis for researchers and clinicians to understand the potential of ML in this field. This study did not examine the effectiveness of individual ML models for predicting PPD. Such research questions would be suitable for future systematic reviews, guided by the framework outlined in our results tables, that is, the effectiveness of specific ML techniques within specific data types for specific clinical applications.

### Conclusions

To conclude, the use of ML to predict PPD has revealed exciting advances, particularly in recent years. Compared with traditional statistical methods, ML algorithms are capable of analyzing larger data sets and performing more advanced computations. Overall, it is clear that ML can significantly improve the detection of PPD at an early stage. Research into the applications of ML to identify potential PPD predictors has demonstrated positive results. However, this work is currently limited, and further research is required to identify additional benefits of ML on maternal mental health. ML techniques and the performance of ML models may differ depending on the type, content, and accuracy of the original data; thus, it may be challenging to evaluate the performance of a single model. With ML tools becoming more accessible to researchers and clinicians, it is expected that the field will continue to grow and that novel applications for mental health will follow. Further clinical research collaborations are required to fine-tune ML algorithms for prediction and treatment. As ML algorithms continue to be refined and improved, it might be possible to help clinicians identify maternal mental illnesses at an earlier stage when interventions may be more effective and personalized treatments based on an individual’s unique characteristics. Moreover, the current lack of procedural evaluation guidelines leaves many clinicians and researchers in the field with no means to systematically evaluate the claims, maturity, and clinical readiness of an ML study [68].

## Appendix

Multimedia Appendix 1 Databases and search strings used for this review.

## Abbreviations

ANN: artificial neural network
AUC: area under the curve
AUROC: area under the receiver operating characteristic curve
DT: decision tree
EHR: electronic health record
ICD: International Classification of Diseases
kNN: k-nearest neighbor
LR: logistic regression
ML: machine learning
NLP: natural language processing
PPD: postpartum depression
PRISMA-ScR: Preferred Reporting Items for Systematic Reviews and Meta-Analyses extension for Scoping Reviews
RF: random forest
SVM: support vector machine
XGBoost: Extreme Gradient Boosting
