# Supplementary material for: Machine Learning Methods for Predicting Postpartum Depression: Scoping Review
Source: JMIR Ment Health. 2021 Nov 24;8(11):e29838. doi: 10.2196/29838 (PMC8663566; doi:10.2196/29838)
Supplement: Multimedia Appendix 1 [file mental_v8i11e29838_app1.docx]

| **Database** | **Strings** | **Results** |
| --- | --- | --- |
| PubMed | (“maternal depression” OR “perinatal depression”) AND (“Baby blues” OR “postpartum blues” *) AND ("machine learning" OR “big data”) AND (“predictive model”) | 198 |
| Scopus | (“maternal Depression” OR “perinatal” OR "postpartum depression”) AND ("machine learning" OR "supervised learning") AND ("predictive model") | 95 |
| ACM digital Library | (“postpartum depression” AND “Machine learning”) OR (“perinatal depression” AND “Big data” OR “artificial intelligence”) | 992 |
| Web of Science | (Depression or Postpartum) AND (“Machine learning” or “Artificial Intelligence”) | 88 |
| PsycInfo | ("postpartum depression" *OR* depression OR pregnancy) AND ("machine learning" *OR* algorithm* OR "artificial intelligence") AND **(**predict* *OR* "predictive model") | 19 |
